# Supplementary material for: Proteolysis Degree of Protein Corona Affect Ultrasound-Induced Sublethal Effects on Saccharomyces cerevisiae: Transcriptomics Analysis and Adaptive Regulation of Membrane Homeostasis
Source: Foods. 2022 Dec 1;11(23):3883. doi: 10.3390/foods11233883 (PMC9735630; doi:10.3390/foods11233883)
Supplement: Supplementary file 1 [file foods-11-03883-s001.zip › foods-1900460-supplementary.pdf]

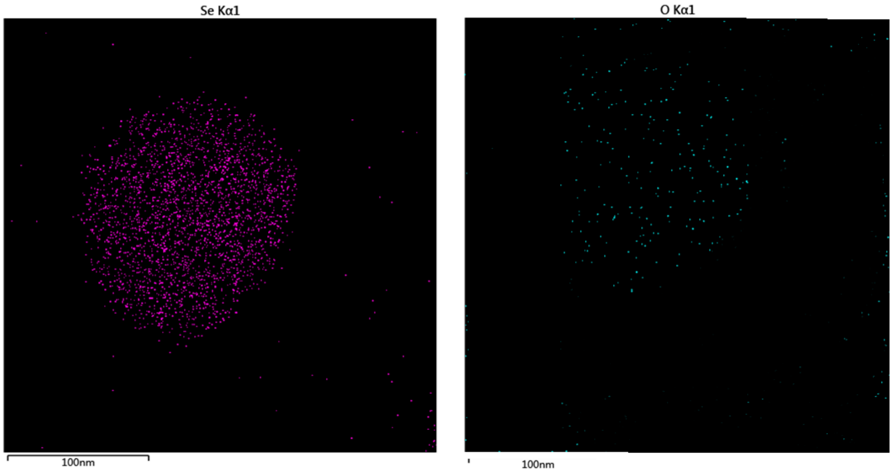

Figure S1. Representative EDS elemental (Se, O) mapping of the prepared nano-Se.

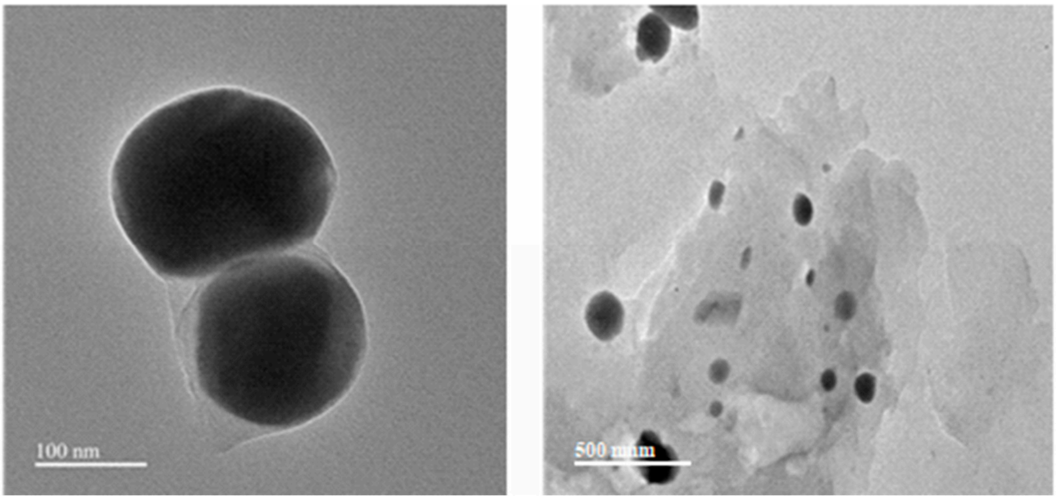

Figure S2. TEM images of the prepared nano-Se.

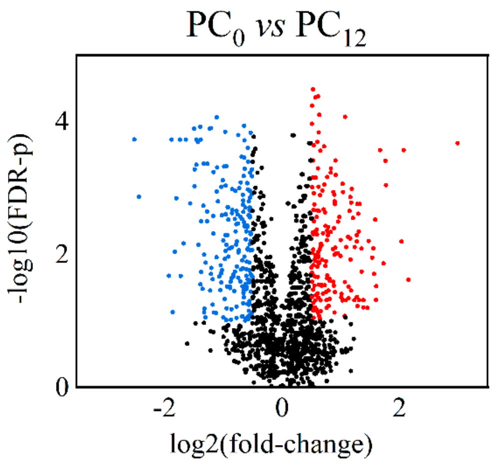

Figure S3. Volcano plots showing the distribution of DEGs between U+nano-Se@PC_0 and U+nano-Se@PC_{12}, arranged by fold-change (x axis) and FDR-p values (y axis). DEGs are colored blue (down-regulation) and red (up-regulation).

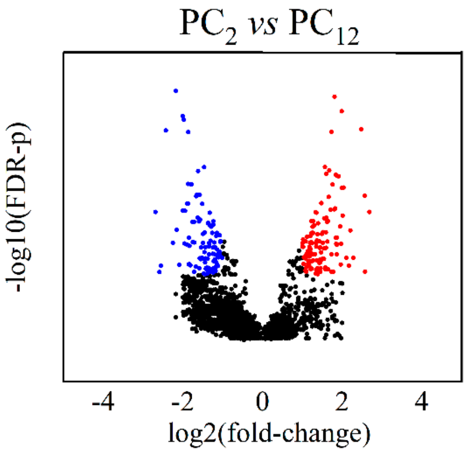

Figure S4. Volcano plots showing the distribution of DEGs between U+nano-Se@PC_2 and U+nano-Se@PC_{12}, arranged by fold-change (x axis) and FDR-p values (y axis). DEGs are colored blue (down-regulation) and red (up-regulation).

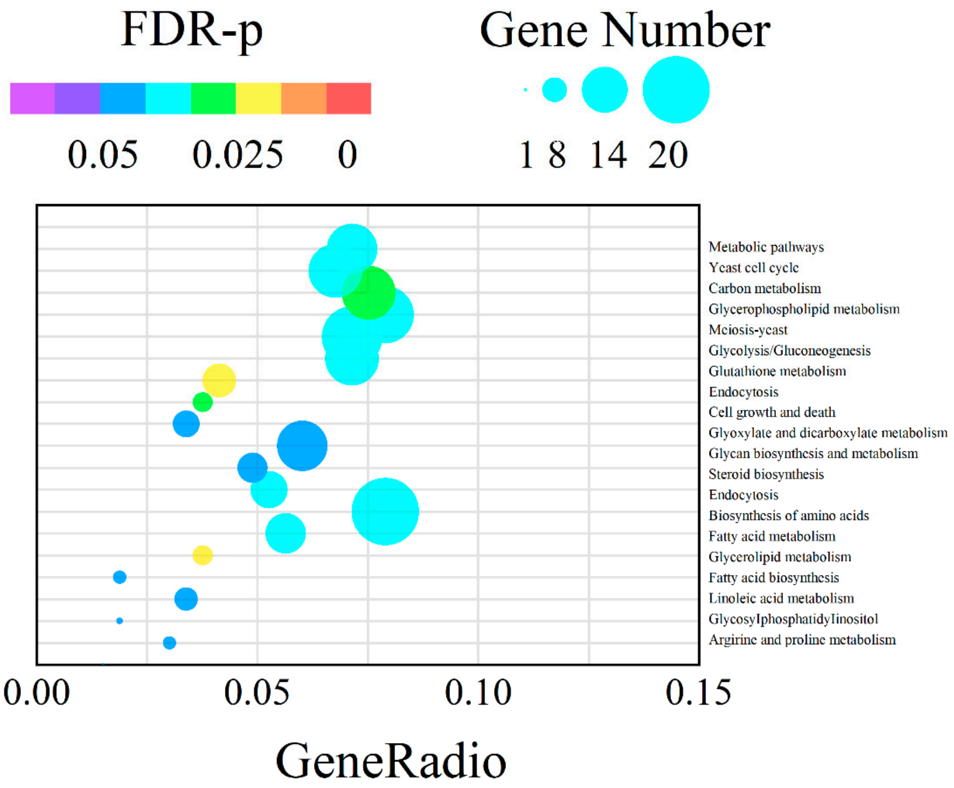

Figure S5. KEGG pathway enrichment analysis of DEGs between U+nano-Se@PC$_2$ and U+nano-Se@PC$_{12}$.

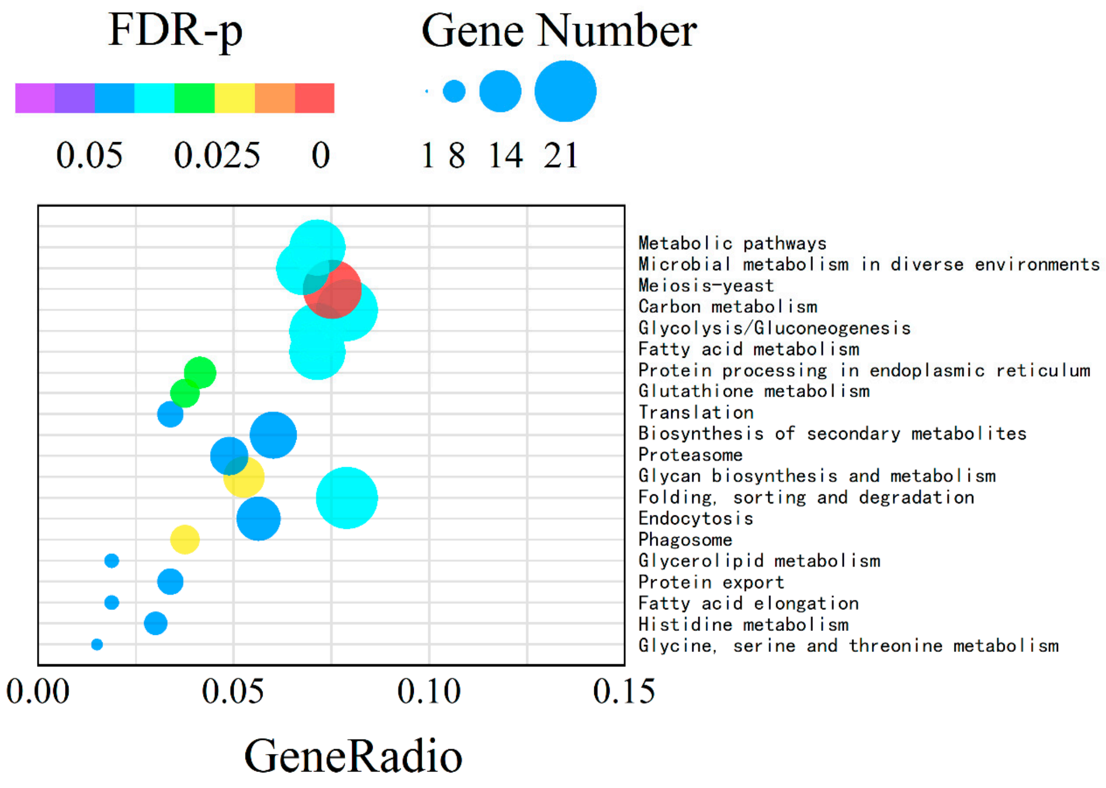

Figure S6. KEGG pathway enrichment analysis of DEGs between U+nano-Se@PC$_0$ and U+nano-Se@PC12.

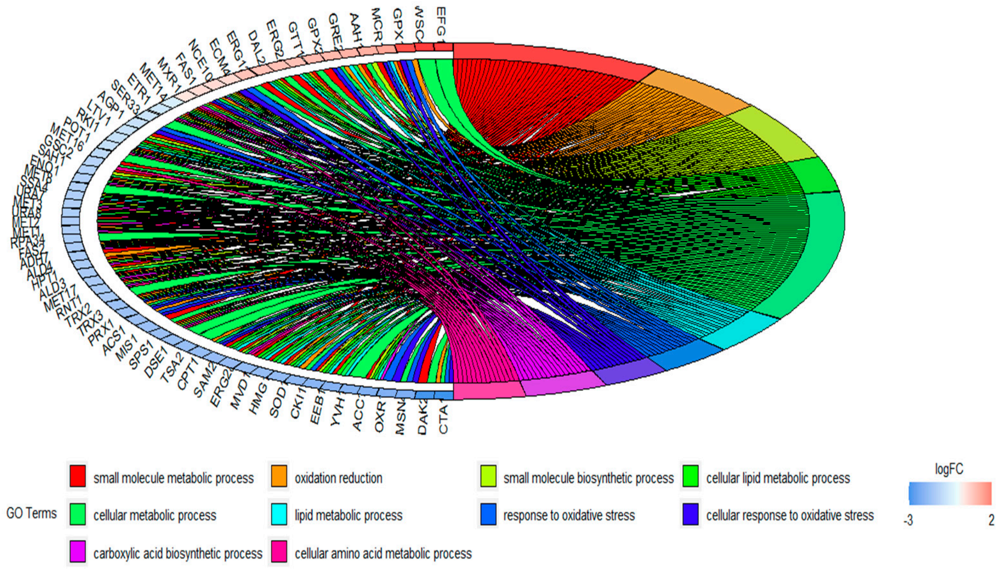

Figure S7. GO classification of DEGs between PC_2 and PC_{12} groups.

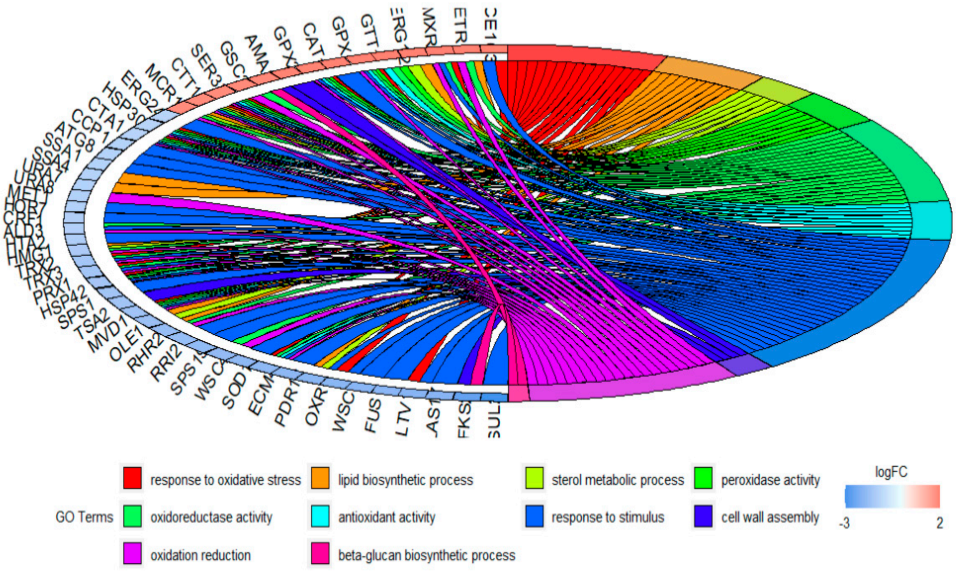

Figure S8. GO classification of DEGs between PC_0 and PC_{12} groups.
